# The impact of wearing a KN95 face mask on human brain function: evidence from resting state functional magnetic resonance imaging

**DOI:** 10.3389/fneur.2023.1102335

**Published:** 2023-05-19

**Authors:** Xiaomeng Wu, Lifei Ma, Qiufeng Yin, Ming Liu, Kyle Wu, Dengbin Wang

**Affiliations:** ^1^Philips (China) Investment Co., Ltd, Shanghai, China; ^2^Department of Radiology, Xinhua Hospital, Shanghai Jiao Tong University School of Medicine, Shanghai, China

**Keywords:** face masks, resting state fMRI, brain function, MRI, brain

## Abstract

**Background:**

Face masks are widely used in daily life because of the COVID-19 pandemic. The objective of this study was to explore the impact of wearing face masks on brain functions by using resting-state functional MRI (RS-fMRI).

**Methods:**

Scanning data from 15 healthy subjects (46.20 ± 6.67 years) were collected in this study. Each subject underwent RS-fMRI scans under two comparative conditions, wearing a KN95 mask and natural breathing (no mask). The amplitude of low frequency fluctuation (ALFF) and functional connectivity under the two conditions were analyzed and then compared using the paired *t*-test.

**Results:**

Compared with those of the no-mask condition, the ALFF activities when wearing masks were increased significantly in the right middle frontal gyrus, bilateral precuneus, right superior marginal gyrus, left inferior parietal gyrus, and left supplementary motor area and decreased significantly in the anterior cingulate gyrus, right fusiform gyrus, left superior temporal gyrus, bilateral lingual gyrus, and bilateral calcarine cortex (*p* < 0.05). Taking the posterior cingulate cortex area as a seed point, the correlations with the occipital cortex, prefrontal lobe, and motor sensory cortex were sensitive to wearing masks compared with not wearing masks (*p* < 0.05). Taking the medial prefrontal cortex region as a seed point, the functional connectivity with the bilateral temporal lobe, bilateral motor sensory cortex, and occipital lobe was influenced by wearing a KN95 mask (*p* < 0.05).

**Conclusion:**

This study demonstrated that wearing a KN95 face mask can cause short-term changes in human resting brain function. Both local neural activities and functional connectivity in brain regions were sensitive to mask wearing. However, the neural mechanism causing these changes and its impact on cognitive function still need further investigation.

## Background

In controlling the global spread of the coronavirus COVID-19, wearing face masks can help control the spread of respiratory fluids and aerosol particles among people to reduce virus transmission. In the daily work of hospitals, medical staff wear masks. When wearing masks, the exhaled air accumulates in the mask and mixes with the inhaled fresh air, resulting in inhalation of more concentrated carbon dioxide (CO_2_). This may lead to mild hypercapnia (1). It is still not clear how the changes caused by wearing face masks might affect brain function. We try to draw a picture of brain functional changes induced by wearing face masks.

Functional magnetic resonance imaging (fMRI) is a sensitive technology to access brain function, and it can show the blood oxygen consumption level of the brain *in vivo* and indirectly reflect the metabolic level changes secondary to brain neural activities. It has been found that brain neural activity could change the contrast of blood oxygen level-dependent (BOLD) signals in both physiological and pathological conditions (2). If wearing a mask causes changes in neural activity, this difference can be detected by fMRI sequencing based on echo planar imaging (EPI).

In recent years, resting-state fMRI (RS-fMRI) has received more attention in studying low-frequency components of BOLD signals and functional connectivity of brain regions. Biswal et al. (3) demonstrated that the human brain has spontaneous neural activities in the RS-fMRI in the absence of goal-directed tasks. This phenomenon was also confirmed by a large number of subsequent studies (4, 5). Acute hypoxia and obstructive sleep apnea hypopnea syndrome lead to changes in spontaneous neural activity of the brain in the resting state (6, 7). Previous studies have shown that changes in the composition of inhaled air flow and the increase of CO_2_ concentration elevated the blood flow in cerebral arteries and affected the blood supply of the cortex (8). RS-fMRI could be an effective method to address the neuro activity changes during hypercapnia induced by wearing masks.

The purpose of this study is to examine the neural functional changes induced by wearing KN95 face masks in the resting state. In this study, comparisons about spontaneous neural activities and functional connectivity were made between the conditions of wearing KN95 face masks and natural breathing without masks. We described the pattern alterations and functional connectivity characters by RS-fMRI data from 15 healthy volunteers. We hypothesize that our findings help to further reveal that mask-wearing hypercapnia could impair neural executive function, attention function, and causes negative emotional experience.

## Materials and methods

### Subjects

This study included 15 healthy subjects (6 males and 9 females), aged from 25 to 48 (46.20 ± 6.67 years). No subjects had any history of neurological disease or diagnosis of schizophrenia, affective disorder, or pervasive development disorder and related family history, and no abnormalities of the central nervous system were found during MRI. This study was reviewed and approved by the ethics committee of Xinhua Hospital affiliated with Shanghai Jiao Tong University School of Medicine, and the informed consent of all subjects was obtained before examination.

### MRI scanning

MRI data were obtained on a Philips Ingenia 3.0 T magnetic resonance scanner (Philips Medical Systems, Best, The Netherlands), with a maximal gradient field strength of 45 mT/m and slew rate of 200 mT/m/ms. A 20-channel standard head coil was used.

**Figure 1:**
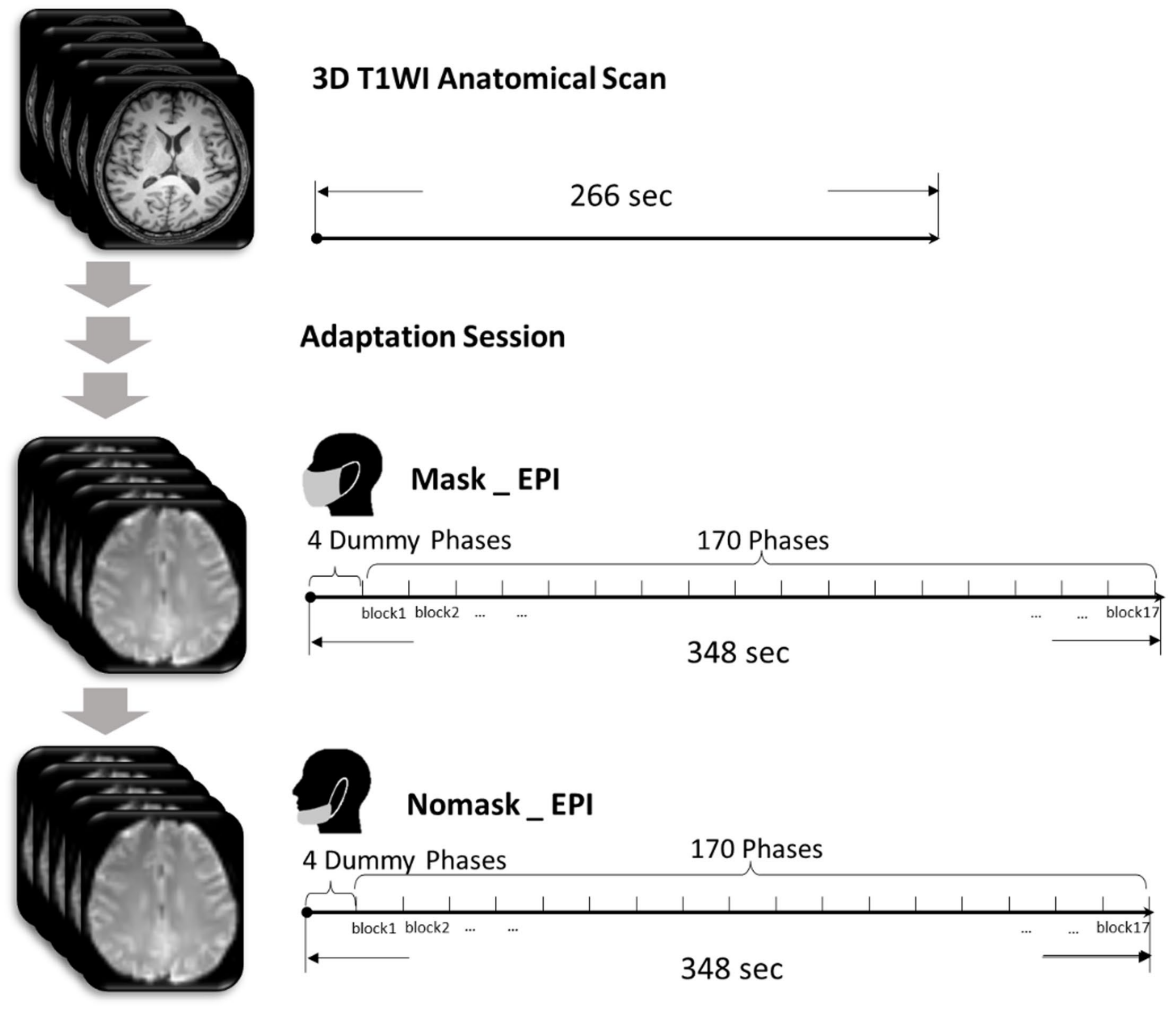
Introduction of experiment design. After T1 weighted anatomical scan and adaptation session, each subject underwent resting-state fMRI scanning under two conditions, wearing a KN95 mask and natural breathing. Conditions with or without face masks were alternated between subjects.

The experiment design was demonstrated in Figure 1. All subjects were scanned in the headfirst, supine position. The fMRI scanning sessions included starting with a three-dimensional T1 weighted anatomical scan, then an additional fMRI adaptation session was carried out before formal scanning for data collection so that the subjects could acclimate to the fMRI examination environment, thereby minimizing the impact of the scanning environment on their resting brain function. Then, the same scanning parameters were used for each subject in functional sessions under two comparative conditions, with and without masks. During the resting-state fMRI sessions, each subject was instructed to remain as still as possible with the head comfortably fixed by foam pads, eyes closed, and no systematic thinking. In the mask-wearing session, a KN95 mask (Zhuhai Gejian Medical Technology, Guangdong, China) covered the subjects’ mouth and nose comfortably and was fixed to both ears by elastic bands. In the session without masks, the subjects pulled down the masks to expose the mouth and nose completely, suspending it on the lower jaw, but masks were not fully removed, and the elastic bands remained fixed on both ears. For each subject, the beginning-to-end experimental time was controlled within 30 min to prevent fatigue. Conditions with or without face masks were alternated between subjects.

T1 weighted anatomy scanning used a three-dimensional gradient echo T1 weighted sequence: repetition time/echo time (TR/TE) = 7.9 ms/3.5 ms, slice thickness 1 mm with gap 0 mm, bandwidth 140.9 Hz/pixel, field of view (FOV) = 250 × 200 × 170 mm^3^, in-plane resolution 1 × 1 mm^2^, and scanning time 4:26 min. The fMRI sessions used a single-shot EPI sequence, TR = 2000 ms, TE = 35 ms, flip angle = 90 degrees, slice thickness 5 mm with gap 0 mm, in-plane resolution 3 × 3 mm^2^, with 27 axial slices covering the whole brain. Bandwidth was 140.9 Hz/pixel, FOV = 240 × 240 mm, and the sequence was composed of 170 dynamics. There were four dummy scans at the beginning of the fMRI sessions to determine the stability of the signal that were not collected. The scanning time was 5:48 min.

### Data processing

The data preprocessing was performed using SPM12^1^, including slice timing, head motion correction (a least squares approach and a six-parameter spatial transformation), and spatial normalization to the Montreal Neurological Institute (MNI) template (resampling voxel size = 3 × 3 × 3 mm^3^). Subsequent data preprocessing included removal of linear trends and spatial smoothing (full width at half maximum = 6 mm Gaussian kernel), followed by a band pass filtered by 0.01 Hz–0.1 Hz. The 3D T1 images were also spatially normalized to the MNI template. The RESTplus toolkit (v. 1.24, http://restfmri.net/forum/restplus) was used to calculate the voxel-wise amplitude of low-frequency fluctuation (ALFF) (9) of each subject as well as region-wise functional connectivity. The signal from cerebrospinal fluid and white matter was regressed out before functional connectivity analysis.

In this study, all measurement data are represented by mean ± SD. Statistical analysis was conducted with the RESTplus toolkit. A one-sample *t*-test was performed within each condition for ALFF and functional connectivity. The mean value between groups was compared by the paired *t*-test (*p* < 0.05 was considered statistically significant).

## Results

**Figure 2:**
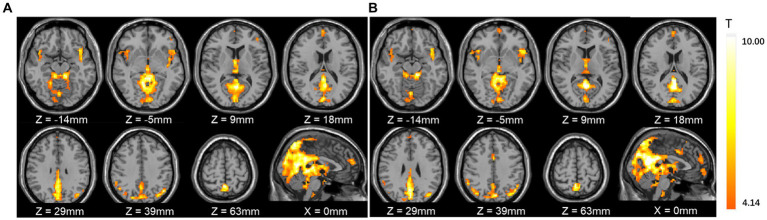
The one sample *t*-test of mean amplitude of low frequency fluctuation (mALFF) activities with and without masks. **(A)** Without masks; **(B)** with masks. The results showed that the mALFF activity patterns were very similar for the two conditions.

**Figure 3:**
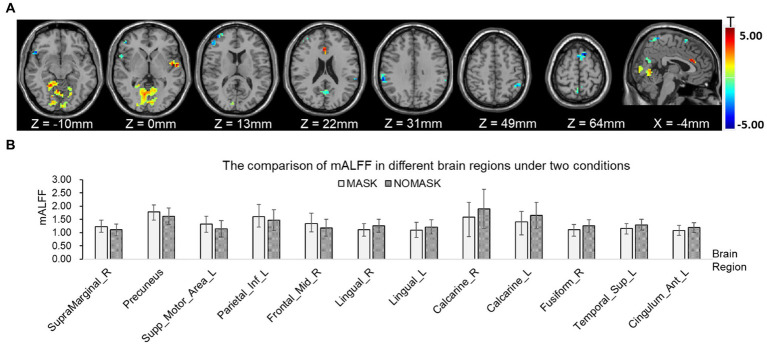
Comparison of mean amplitude of low frequency fluctuation (mALFF) with and without masks by paired *t*-tests. **(A)** mALFF activities in brain regions were significantly different with and without face masks. **(B)** Comparison of power spectrum of mALFF in different brain regions. The posterior cingulate cortex (PCC, MNI coordinates: *x* = 0 mm, *y* =−45 mm, *z* = 27 mm, radius 6 mm) and medial prefrontal cortex (MPFC, MNI coordinates: *x* = 0 mm, *y* = 63 mm, *z* = 13 mm, radius 6 mm) were selected as seed points for functional connectivity analysis. The results of the one-sample *t*-test with and without face masks showed that the PCC was positively correlated with the precuneus, middle temporal lobe, inferior temporal lobe, medial prefrontal lobe, and orbital inferior frontal gyrus and negatively correlated with the superior marginal gyrus, middle cingulate cortex, rolandic operculum, precentral lobe, postcentral lobe, and fusiform gyrus. The MPFC was positively correlated with the precuneus, posterior cingulate gyrus, supplementary motor cortex, angular gyrus, middle temporal lobe, and inferior temporal lobe and negatively correlated with the superior marginal gyrus, middle cingulate cortex, precentral lobe, postcentral lobe, middle occipital cortex, middle frontal gyrus, and inferior frontal gyrus of the triangle (*p* < 0.05, Figure 4).

The one sample *t*-test analysis of the mean amplitude of low frequency fluctuation (mALFF), for each condition in the resting state was shown in Figure 2. The brain regions of ALFF activities were mainly significant in the posterior cingulate gyrus, precuneus, medial prefrontal cortex (MPFC), and other brain regions (*p* < 0.001) with or without a face mask. A paired *t*-test was then conducted to compare conditions with and without masks. The results showed that compared with those without masks, in those with masks the mALFF activities in the right middle frontal gyrus, bilateral precuneus, right superior marginal gyrus, left inferior parietal gyrus, and left supplementary motor area increased significantly. The signals in anterior cingulate gyrus, right fusiform gyrus, left superior temporal gyrus, bilateral lingual gyrus, and bilateral calcarine cortex decreased significantly (*p* < 0.05, see Table 1; Figure 3).

**Table 1:** The comparison of mean amplitude of low frequency fluctuation (mALFF) activity difference with and without masks (p < 0.05, cluster size ≥ 540 mm^3^, Alfa SIM corrected).

Brain region	BA	Volume (mm^3^)	MNI coordinates			*T* value	*Z* value
			*X*	*Y*	*Z*		
Mask > no mask							
SupraMarginal_R	40	1,242	63	−36	33	4.94	3.70
Bilateral precuneus	23	1,242	−3	−69	27	4.61	3.54
Supp_Motor_Area_L	8	1,053	−12	15	63	5.04	3.74
Parietal_Inf_L	40	1,053	−48	−48	51	3.62	2.99
Frontal_Mid_R	46	621	39	54	15	4.09	3.26
Mask < no mask							
Lingual_R	18	3,699	17	−74	−13	4.66	3.56
Lingual_L	18	3,132	−19	−75	−11	4.74	3.60
Calcarine_R	17	2,808	9	−78	3	3.29	2.79
Calcarine_L	17	2,295	−7	−86	−1	3.26	2.77
Fusiform_R	37	1,323	27	−54	−15	6.52	4.35
Temporal_Sup_L	48	972	−48	−6	−3	5.62	4.00
Cingulum_Ant	24	594	2	24	25	4.51	3.49

**Figure 4:**
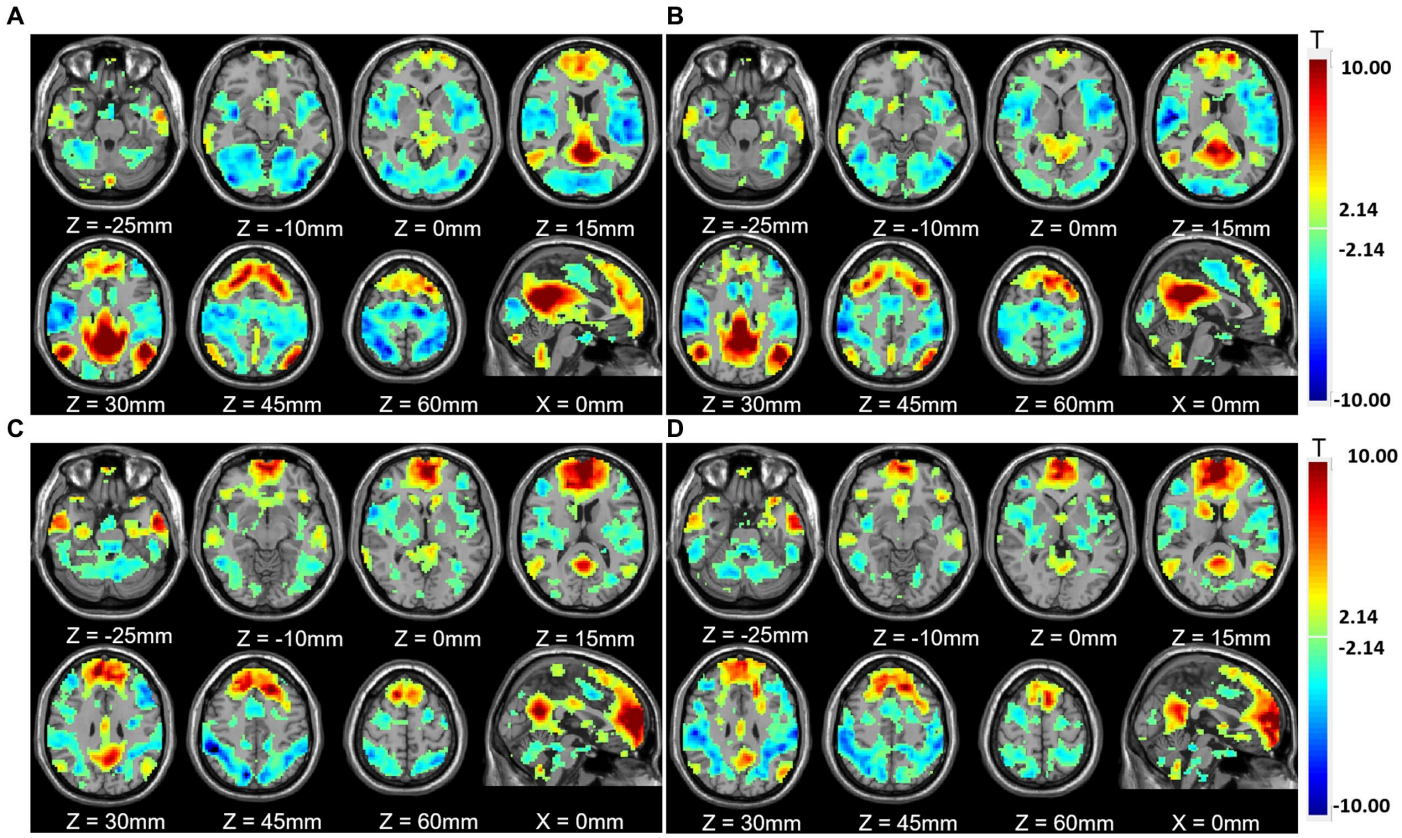
One-sample *t*-test results of functional connectivity for each condition. **(A)** Functional connectivity of posterior cingulate cortex (PCC) without mask. **(B)** Functional connectivity of PCC with mask. **(C)** Functional connectivity of the medial prefrontal cortex (MPFC) without mask. **(D)** Functional connectivity of the MPFC with mask.

**Figure 5:**
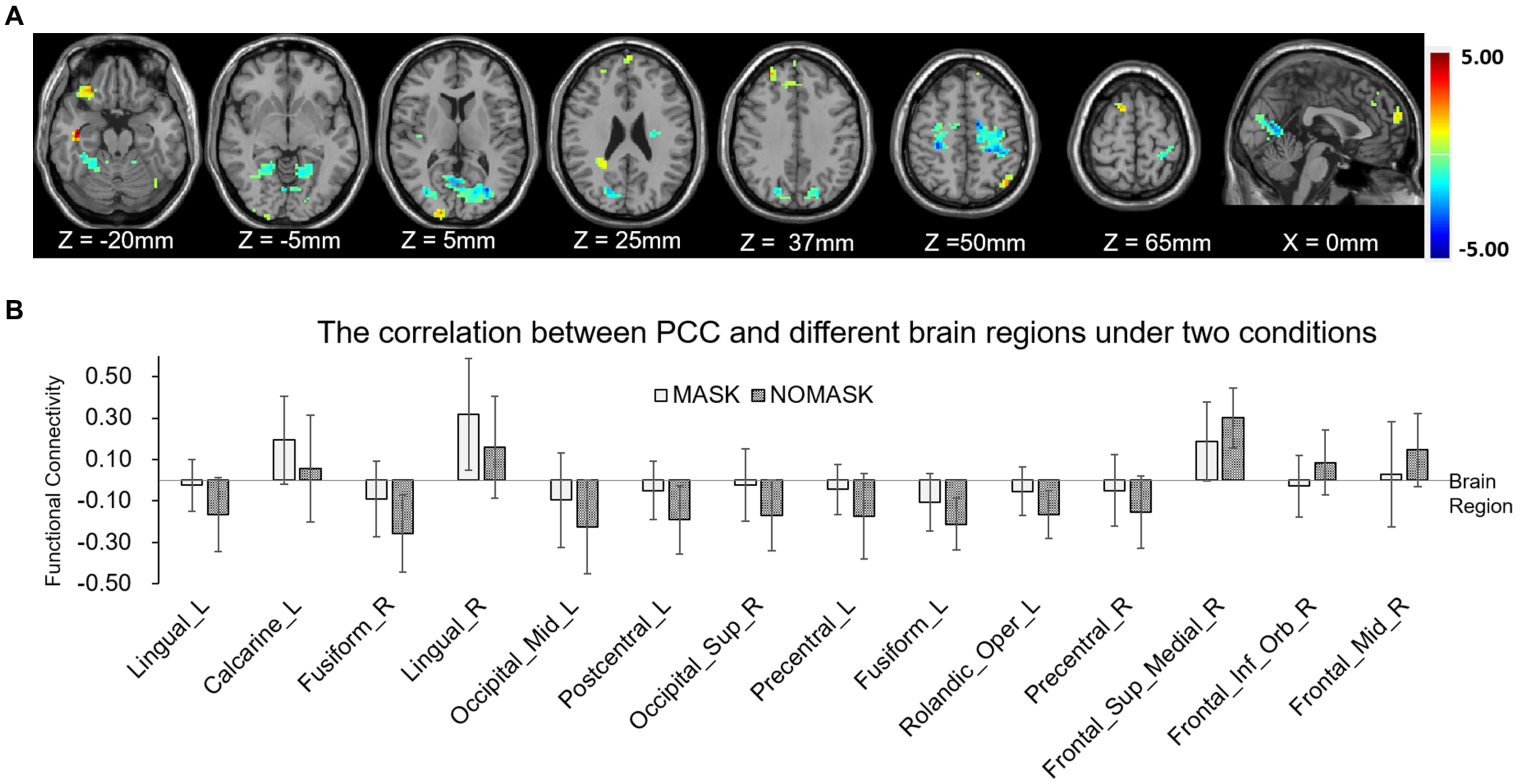
Functional connectivity comparison based on posterior cingulate cortex (PCC) correlation with whole brain activity with and without masks. **(A)** Brain regions in which the functional connectivity were significantly different with and without face masks. **(B)** Comparison of correlation strength in different brain regions with and without masks.

**Figure 6:**
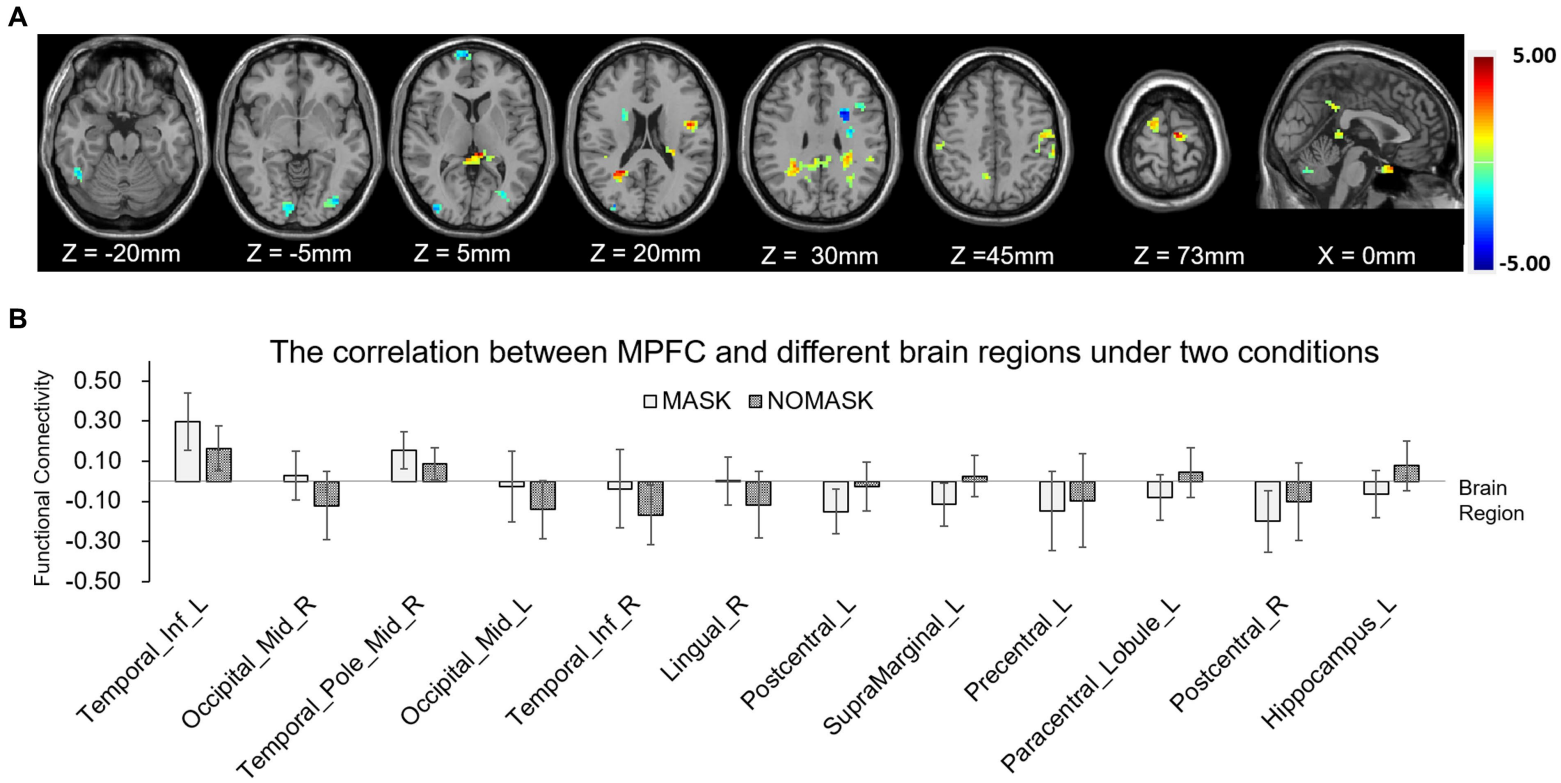
Functional connectivity comparison based on the medial prefrontal cortex (MPFC) correlation with whole brain activity with and without masks. **(A)** Brain regions in which functional connectivity were significantly different with and without face masks. **(B)** Comparison of correlation strength in different brain regions with and without masks.

The paired *t*-test results showed that compared with those without masks, the positive correlation between the PCC and the cortex around the left calcarine cortex and the right lingual gyrus was enhanced (see Table 2). The positive correlation with medial prefrontal lobe and right middle frontal gyrus decreased. The negative correlation with left lingual gyrus, bilateral fusiform gyrus, left middle occipital gyrus, right superior occipital gyrus, left postcentral gyrus, bilateral precentral gyrus, and left rolandic operculum decreased (Figure 5). The positive correlation between the MPFC and left inferior temporal gyrus and right middle temporal gyrus was enhanced. The negative correlation with bilateral posterior central gyrus and left anterior central gyrus was enhanced. The negative correlation with left middle occipital gyrus, right inferior temporal gyrus, and right lingual gyrus was weakened (Figure 6).

**Table 2:** The comparison of functional connectivity differences with and without masks (*p* < 0.05, cluster size ≥ 540 mm^3^, Alfa SIM corrected).

Brain region	BA	Volume (mm^3^)	MNI coordinates			*T* value	*Z* value
			*X*	*Y*	*Z*		
PCC							
**Mask > no mask**							
Lingual_L	18	4,428	−19	−83	−1	4.03	3.23
Calcarine_L	17	3,726	−4	−70	11	3.82	3.11
Fusiform_R	37	3,429	31	−48	−12	3.44	2.88
Lingual_R	17	3,213	2	−64	9	3.24	2.75
Occipital_Mid_L	19	3,213	−30	−75	12	5.03	3.74
Postcentral_L	3	2,673	−30	−33	48	5.45	3.93
Occipital_Sup_R	18	1809	20	−74	20	3.66	3.01
Precentral_L	6	1,620	−34	−12	49	3.82	3.11
Fusiform_L	37	1,242	−28	−51	−11	2.83	2.47
Rolandic_Oper_L	41	729	−39	−36	15	3.68	3.02
Precentral_R	3	594	30	−27	51	4.44	3.44
**Mask < no mask**							
Frontal_Sup_Medial	10	1,269	0	63	21	3.23	2.74
Frontal_Inf_Orb_R	47	729	27	27	−18	4.93	3.69
Frontal_Mid_R	46	729	27	48	30	4.61	3.53
MPFC							
**Mask > no mask**							
Temporal_Inf_L	20	1,539	−54	−6	−39	3.94	3.18
Occipital_Mid_R	19	1,512	39	−87	12	5.03	3.74
Temporal_Pole_Mid_R	20	837	39	18	−42	3.64	3.00
Occipital_Mid_L	19	756	−39	−81	−6	3.40	2.85
Temporal_Inf_R	37	702	48	−51	−21	3.26	2.76
Lingual_R	18	594	9	−87	−6	3.07	2.64
**Mask < no mask**							
Postcentral_L	3	2,133	−60	−15	39	3.75	3.06
SupraMarginal_L	48	754	−51	−38	30	2.71	2.38
Precentral_L	6	609	−46	−3	23	4.13	3.28
Paracentral_Lobule_L	6	540	−6	−12	75	7.07	4.54
Postcentral_R	3	540	51	−18	39	3.47	2.90
Hippocampus_L	27	540	−17	−35	7	2.42	2.18

## Discussion

This study aims to investigate the effect of wearing a mask on the brain function，and alterations in regional neural activity were observed while wearing masks. Considering that the ever-changing experimental operation and the complex neural functions involved, it was very difficult to comprehensively understand the changes of brain function when wearing masks for only a short time. Thus, resting-state but not goal-directed fMRI was used in this study. In fact, previous studies by Law and Klugah-Brown et al. did not find significant changes in brain function caused by wearing masks with task-fMRI (10, 11). In the present study, RS-fMRI was very effective for investigating the regional and overall functional differences in the brain in mild hypercapnia and for clarifying the changes in brain function caused by wearing face masks.

In this study, ALFF signal changes in brain function was obvious when wearing masks. The results of ALFF showed that in the resting state, the posterior cingulate gyrus, precuneus, anterior cingulate gyrus, and medial prefrontal lobe in the midline structure of the brain showed higher spontaneous neural activity. This was likely to be related to high CO_2_ concentration in inhaled air flow caused by wearing a mask. A recent study found that although wearing a mask increased the partial pressure of CO_2_ at the end-tidal air flow of respiration up to 7.4% (10). In their results, hypercapnia caused by mild CO_2_ storage was shown to increase the regional blood perfusion of the brain, resulting in the elevation of the BOLD signal baseline. However, because of the limited sample size, they may not have been able to show the subtle differences in neural activities between wearing and un-wearing masks. Previous research also found that, regional cerebral blood flow decreased in DMN but increased in other regions in healthy adults during hypoxia (12). In our study, it was found that the effect of wearing a mask or not on DMN function was inconsistent across different brain regions. This suggested that under hypoxia, compensatory oxygen metabolism in specific brain regions of the DMN increases to ensure fundamental neural functions, even though overall perfusion in DMN was decreased. In the resting state (awake, eyes closed), the brain regions of the DMN had synchronous and consistent activities, maintaining an alert state in the brain and continuously evaluating sensory input and the surrounding environment (3, 13). The PCC, precuneus and MPFC in the DMN played an important role to maintain human cognitive function, and their dysfunction could even be used as a warning signal of cognitive impairment in Alzheimer’s disease (14).It was reported that hypoxic exposure may lead to cognition dysfunction in healthy adults (15–17), which was likely related to the signal changes of DMN found in this work. The extensive changes of DMN signal in this study may suggest the effect of wearing masks on brain function like attention alert and self-evaluation.

The results of this study also indicated that wearing masks may further affect functional connectivity between brain regions such as synergism or antagonism. In this study, PCC and MPFC of DMN were used as seed points, and functional connectives between PCC and visual network (occipital lobe), dorsal attention network (precentral gyrus, orbital inferior frontal gyrus), as well as MPFC and visual network (occipital lobe), central executive network (temporal lobe) were significantly different under those two conditions. This difference suggested that the hypoxic environment may alter the patterns of interaction among large-scale brain networks compared to normal ventilation status, resulting in broad changes in the neuro function. This founding was consistent with the results of RS-fMRI in previous studies on acute hypoxia and obstructive sleep apnea hypopnea syndrome (6, 7). Similar effect was also reported in a recent study, Haller et al. found that wearing masks may change the patterns functional connectivity with the salience network (18). The mechanism behind this was still unclear, we speculated that the partial reason of changes in functional connectivity may be related to the dyspnea caused by KN95 mask and the resulting adverse emotional experience. The functional connectivity in people with depression and anxiety was verified as differing from that of normal people (19–21). A recent fMRI study about Crohn’s disease also indicated that the functional connectivity between the PCC and precuneus was associated with emotional experience induced by recurrence and chronic process of disease (19). Though findings on changes in functional connectivity stability were not consistent among various studies, PCC and MPFC were generally involved in subjective assessment and emotional processing.

Some limitations should be considered in this study. Firstly, the findings, based on 15 healthy volunteers, still need to be confirmed in broader data. Secondly, some preliminary results were obtained with RS-fMRI, but whether wearing a mask will lead to brain dysfunction during the execution of tasks still needs to be explored. Finally, the short-term effect of wearing masks was reported in this and other study, whether wearing a mask for a long time would aggravate such changes has not been further explored. Given the current global situation, the effects of long-term mask-wearing were worth further investigation.

## Conclusion

In this study, the RS-fMRI technique was used to show the potential impact on neural function in normal people wearing KN95 masks. The results showed that wearing KN95 masks could change spontaneous neural activity and the functional connectivity patterns in the brain. These results suggest that people who need to wear masks in their daily work should appropriately increase the rest time or shorten the work cycle. If the rest conditions cannot be met, it is recommended to wear forced ventilation masks to avoid any problems caused by changes in brain function.

## Data availability statement

The original contributions presented in the study are included in the article/supplementary material, further inquiries can be directed to the corresponding author.

## Ethics statement

The studies involving human participants were reviewed and approved by The Institutional Ethics Committee of Xinhua Hospital affiliated to Shanghai Jiao Tong University School of Medicine. The patients/participants provided their written informed consent to participate in this study.

## Author contributions

DW is the guarantor of the manuscript and takes responsibility for the integrity of the work as a whole, from inception to published article. QY, ML, XW, LM, KW, and DW conceived the study concept and design and contributed to critical revision of the manuscript. QY, ML, XW, LM, and KW performed the acquisition of data and conducted data analysis and interpretation. QY, ML, and LM drafted the manuscript. All authors contributed to the article and approved the submitted version.

## Funding

This work was supported by Science and Technology Commission of Shanghai Municipality [no. 19411951402]. The funders had no role in study design, data collection and analysis, decision to publish, or preparation of the manuscript.

## Conflict of interest

Authors XW, LM, and KW were employed by Philips (China) Investment Co., Ltd.

The remaining authors declare that the research was conducted in the absence of any commercial or financial relationships that could be construed as a potential conflict of interest.

## Publisher’s note

All claims expressed in this article are solely those of the authors and do not necessarily represent those of their affiliated organizations, or those of the publisher, the editors and the reviewers. Any product that may be evaluated in this article, or claim that may be made by its manufacturer, is not guaranteed or endorsed by the publisher.

## Footnotes

^1^ http://www.fil.ion.ucl.ac.uk/spm

## Abbreviations

COVID-19: coronavirus disease 2019
RS-fMRI: resting-state fMRI
BOLD: blood oxygen level dependent
mALFF: mean amplitude of low-frequency fluctuation
MNI: Montreal Neurological Institute
PCC: posterior cingulate cortex
MPFC: medial prefrontal cortex
DMN: default mode network.
